# INITIAL DEVELOPMENT OF THE WRITING TO HEAL APP: A STRUCTURED WRITING SERIES FOR CAREGIVERS OF SPOUSES LIVING WITH ADRD

**DOI:** 10.1093/geroni/igae098.4368

**Published:** 2024-12-31

**Authors:** Angie LeRoy, Samantha Weiss, Katherine Harris, Valentina Maza, Sierra Wickline, Alexandria Henderson, Leona Liskovec

**Affiliations:** University of North Florida, Jacksonville, Florida, United States; Baylor University, Waco, Texas, United States; Baylor University, Waco, Texas, United States; Rice University, Waco, Texas, United States; University of North Florida, Jacksonville, Florida, United States; Baylor University, Waco, Texas, United States; Third i Research & Design, Tallahassee, Florida, United States

## Abstract

Caregiving for a spouse living with Alzheimer’s or a Related Dementia (ADRD) is incredibly stressful, and puts caregivers at risk for developing health problems, themselves. Caregivers’ health issues may affect not only their own quality of life but also their ability to care for their spouse. Thus, developing interventions that support caregivers and help mitigate the stress associated with caregiving, is critical. In Part I of the study, we conducted focus groups with people caring for a spouse living with ADRD) to identify opportunities for targeted treatment and potential barriers to a cognitive-based online expressive writing intervention specifically tailored to spousal caregivers. Results from Part I indicated that caregivers had dynamic needs and requested an intervention that was efficient, mobile, and readily accessible. Collaborating with a product designer to review the qualitative results from Part I, the research team determined that an app would be the best-fitting modality for intervention delivery. In Part IIa, we conducted a second wave of focus groups, throughout which we iteratively adapted the design for an app-based writing intervention for stress and grief among spousal caregivers. To prepare for app development, we conducted preliminary usability testing (Part IIb), during which caregivers interacted with a prototype of the future app to complete a number of proposed task flows. In this article, we present methods and results from Parts I & II as well as reviewing our future plans for app development and subsequent beta testing of the intervention in a pilot RCT.

